# Sub-optimal pH Preadaptation Improves the Survival of *Lactobacillus plantarum* Strains and the Malic Acid Consumption in Wine-Like Medium

**DOI:** 10.3389/fmicb.2017.00470

**Published:** 2017-03-22

**Authors:** Mariantonietta Succi, Gianfranco Pannella, Patrizio Tremonte, Luca Tipaldi, Raffaele Coppola, Massimo Iorizzo, Silvia Jane Lombardi, Elena Sorrentino

**Affiliations:** Department of Agricultural, Environmental and Food Sciences (DiAAA), University of MoliseCampobasso, Italy

**Keywords:** wine, malolactic fermentation, cross-resistance, ethanol, pH

## Abstract

Forty-two oenological strains of *Lb. plantarum* were assessed for their response to ethanol and pH values generally encountered in wines. Strains showed a higher variability in the survival when exposed to low pH (3.5 or 3.0) than when exposed to ethanol (10 or 14%). The study allowed to individuate the highest ethanol concentration (8%) and the lowest pH value (4.0) for the growth of strains, even if the maximum specific growth rate (μ_*max*_) resulted significantly reduced by these conditions. Two strains (GT1 and LT11) preadapted to 2% ethanol and cultured up to 14% of ethanol showed a higher growth than those non-preadapted when they were cultivated at 8% of ethanol. The evaluation of the same strains preadapted to low pH values (5.0 and 4.0) and then grown at pH 3.5 or 3.0 showed only for GT1 a sensitive μ_*max*_ increment when it was cultivated in MRS at pH 3 after a preadaptation to pH 5.0. The survival of GT1 and LT11 was evaluated in Ringer's solution at 14% ethanol after a long-term adaptation in MRS with 2% ethanol or in MRS with 2% ethanol acidified at pH 5.0 (both conditions, BC). Analogously, the survival was evaluated at pH 3.5 after a long-term adaptation in MRS at pH 5.0 or in MRS BC. The impact of the physiologic state (exponential phase *vs* stationary phase) on the survival was also evaluated. Preadapted cells showed the same behavior of non-preadapted cells only when cultures were recovered in the stationary phase. Mathematical functions were individuated for the description of the survival of GT1 and LT11 in MRS at 14% ethanol or at pH 3.5. Finally, a synthetic wine (SW) was used to assess the behavior of *Lb. plantarum* GT1 and LT11 preadapted in MRS at 2% ethanol or at pH 5.0 or in BC. Only GT1 preadapted to pH 5.0 and collected in the stationary phase showed constant values of microbial counts after incubation for 15 days at 20°C. In addition, after 15 days the L-malic acid resulted completely degraded and the pH value increased of about 0.3 units.

## Introduction

Useful microorganisms or antimicrobial natural substances are commonly used in the manufacture of various fermented products to ensure a more controllable fermentation, to shorten the ripening, to improve the safety or to enhance the flavor (Tremonte et al., 2007, 2010, 2016a; Sorrentino et al., 2013; Di Luccia et al., 2016). Specifically, in winemaking processes some lactic acid bacteria (LAB) are able to perform the malolactic fermentation (MLF), a desirable transformation occurring by means of the malolactic enzyme, constitutive only in some LAB species naturally selected during the alcoholic fermentation (Lonvaud-Funel, 1999). MLF is a biological deacidification characterized by the decarboxylation of tricarboxylic L-malic acid to dicarboxylic L-lactic acid and CO2 (Lerm et al., 2010; Testa et al., 2014). Oenococcus oeni is the main LAB species frequently isolated at the end of the fermentation process and its ability to survive the harsh conditions of wine and to perform the malolactic transformation are the main important features for the use in commercial starter cultures for winemaking. However, other LAB species, mainly *Lactobacillus plantarum*, are frequently found in wine (Cañas et al., 2009; Ruiz et al., 2010; Iorizzo et al., 2016). Strains belonging to this species are widely diffused and often isolated from different environmental niches such as grape, must, wine, dairy, bakery, and probiotic products (Beneduce et al., 2004; Ribéreau-Gayon et al., 2006; Cañas et al., 2009; Ruiz et al., 2010; Reale et al., 2011, 2013; Ricciardi et al., 2014; Succi et al., 2014; Testa et al., 2014). In wine, apart from the aptitude to cope with stress conditions, mainly represented by high alcohol concentration and low pH (Spano and Massa, 2006; López et al., 2008; Miller et al., 2011), some *Lb. plantarum* strains are also able to perform the MLF. Moreover, several strains belonging to this species hold enzymes encoding important genes (e.g., citrate lyase, phenolic acid decarboxylase, esterase) for the production of wine aroma compounds (Matthews et al., 2004; Spano et al., 2005; Mtshali et al., 2010; duToit et al., 2011), thus being considered as the most interesting candidate to act as starter cultures in winemaking.

*Lb. plantarum*, due to the facultatively heterofermentative properties, is homo-fermentative for hexoses, which decreases the risk of acetic acid production and the consequent increase in the volatile acidity of the wine (Lonvaud-Funel, 1999; Ribéreau-Gayon et al., 2006). For this reason, it can be also recommended for coinoculation with yeasts when used in the presence of sugars (duToit et al., 2011).

Moreover, the market offers a mixed formulation for MLF, which consists in a blend of *O. oeni* and *Lb. plantarum*, assuring the winemaker an optimal fermentation course.

The ability of *Lb. plantarum* to survive to specific stress factors encountered in wine (e.g., acid pH, cold), as well as other stress factors in various foods (e.g., bile, osmotic, heat, high pressure) was widely explored (van de Guchte et al., 2002; De Angelis and Gobbetti, 2011). Moreover, in recent years some Authors (Bravo-Ferrada et al., 2013, 2014, 2015, 2016) reported the positive effect of acclimation to ethanol concentrations lower than that of wine on the viability and malic acid consumption of oenological *Lb. plantarum* strains. Other studies (Brizuela et al., 2017) showed that no pre-acclimation treatment at sub-lethal ethanol concentration was required for Patagonian *Lactobacillus plantarum* strains used in winemaking.

However, to our knowledge no study reported the effect of preadaptation to low pH on the survival and MLF of *Lactobacillus plantarum*. For this reason, the present work was planned to investigate the effect of preadaptation conditions, in terms of pH and ethanol, on the ability of oenological *Lactobacillus plantarum* to survive and to perform MLF in wine-like medium.

## Materials and methods

### Screening assay

Forty strains of *Lb. plantarum*, previously isolated from traditional red wines and available in the culture collection of the DiAAA (Dept. of Agricultural, Environmental and Food Science, University of Molise), were screened in order to assess the cell survival under acid or ethanol stress conditions. The commercial strain *Lb. plantarum* v22 (Lallemand Inc., Montreal, Canada) and the type strain *Lb. plantarum* DSMZ 20174 (Leibniz Institute DSMZ-German Collection of Microorganisms and Cell Cultures, Braunschweig, Germany) were used as controls. Strains, stored at −80°C in Skim Milk (Succi et al., 2007), were propagated twice in MRS broth at 28°C prior their use. Then, 50 mL of each culture, grown in MRS broth (Oxoid, Milan, Italy) at 28°C, were taken in the mid-exponential phase (OD_600_ = 2–3), standardized at an OD_600_ = 2 (corresponding to 1 × 10^9^ CFU/mL) and centrifuged at 7,500 rcf for 15 min at 4°C. The pellet was washed 2 times with 1X phosphate buffer (1X PBS) and resuspended in 50 mL sterile Ringer's solution (RS) (Oxoid, Milan, Italy) containing ethanol (10 or 14%, v/v), or acidified with HCl up to pH 3.5 or 3.0. Inoculated broths were incubated for 2 h at 28°C and the viable count was performed in order to assess the cell survival. The results were expressed as Ln (N/N_0_), where N are the CFU/mL after 2 h of incubation and N_0_ are the CFU/mL at time 0.

At the end of the screening assay, 10 strains were selected on the basis of their different response to ethanol or acid stress and they were used in the subsequent experiments.

### Effect of ethanol and low pH on the growth of *Lb. plantarum*

Batch fermentations were carried out at 28°C in Erlenmeyer flaks containing 500 mL of MRS broth containing 2, 4, 8, 10, or 14% (v/v) of ethanol. For this purpose, 1% of each overnight culture was inoculated into sterile MRS broth added with filter-sterilized ethanol (Filter Unit Red 0.22-μm pore size; Schleider & Schuell, Dassel, Germany) at different concentrations. A fermentation in MRS broth without ethanol was performed as control for each strain. Microbial growth was followed over the time by measuring the optical density at 600 nm (OD_600_). The maximum specific growth rate (μ_*max*_) was calculated by linear regression of Ln (OD/OD_0_) as a function of the time, where OD_0_ is the optical density at the beginning of the exponential growth phase.

Similarly, the effect of low pH on the growth of *Lb. plantarum* strains was assessed. In short, overnight cultures (1%) were inoculated in Erlenmeyer flaks containing 500 mL of sterile MRS broth acidified with HCl until pH 3.0, 3.5, 4.0, 4.5, 5.0, or 5.5. A fermentation in MRS broth at pH 6.5 was performed as control for each strain. The microbial growth was followed over time as reported above.

### Effect of long-term adaptation to ethanol and low pH on the growth of *Lb. plantarum*

Strains of *Lb. plantarum*, cultivated in MRS broth containing 2 and 8% of ethanol as described above, were transferred (1%) at the beginning of the stationary phase in MRS broth containing 8, 10, or 14% of ethanol. A fermentation in MRS broth without ethanol was performed as control for each strain.

Similarly, strains cultivated in MRS broth at pH 4.0 and 5.0 as described above, were collected by centrifugation at the beginning of the stationary phase, and transferred (1%) in MRS broth acidified at pH 3.5 or 3.0 with HCl. A fermentation in MRS broth at pH 6.5 was performed as control for each strain. Microbial growth was monitored over time by measurement of the optical density at 600 nm (OD_600_) of the cultures. For each experiment, the microbial growth and the maximum specific growth rate (μ_*max*_) were obtained as described previously.

### Effect of sub-optimal pH and ethanol concentration on the growth of *Lb. plantarum*

Two strains of *Lb. plantarum* (GT1 and LT11) were cultivated at 28°C in MRS broth containing 2% of ethanol, in MRS broth at pH 5.0, both prepared as reported above, and in MRS broth containing 2% of ethanol and acidified at pH 5.0 (both conditions, BC). MRS at pH 6.5 without alcohol was used as control. The microbial growth was estimated over time by measuring the optical density at 600 nm (OD_600_) and reported as Ln (OD/OD_0_), where OD_0_ is the optical density at the beginning of the exponential growth phase.

### Effect of long-term adaptation to ethanol and low pH on the survival of *Lb. plantarum*

*Lb. plantarum* GT1 and LT11 were cultivated at 28°C in MRS broth containing 2% of ethanol, in MRS broth at pH 5.0 and in MRS broth BC. MRS at pH 6.5 without alcohol was used as control. In the middle of the exponential growth phase or at the beginning of the stationary phase, cultures were centrifuged (7,500 rcf for 15 min at 4°C), the supernatant was discarded and the pellet was washed twice with a phosphate buffered saline (PBS). Cellular pellet was suspended (about 3.0 × 10^8^ CFU/mL) in RS containing 14% of ethanol or in RS acidified at pH 3.5 and incubated for 24 h at 28°C. At regular time intervals, an aliquot of cultures was recovered and enumerated by plate counts on MRS agar (Oxoid). Plates were incubated at 28°C for 72 h under anaerobic conditions using an anaerobic system (Oxoid). Three replicates were made for each experiment.

### Fitting of survival data

The log-transformed survival data were modeled using the linear or the non-linear regression approach. For this purpose, the Geeraerd and Van Impe Inactivation Model Fitting Tool (GInaFiT) was used, comprising nine different models (Geeraerd et al., 2005). In particular, the biphasic-linear model (Cerf, 1977) Equation 1, the Log-linear model with shoulder and tail (Geeraerd et al., 2000) Equation 2, and the double Weibull model (Coroller et al., 2006) Equation 3 are reported in the following formulae.

(1) $${\text{log}}_{10}(N)={\text{log}}_{10}(N_{0})+{\text{log}}_{10}(f.e^{-k_{max1}.t}+(1-f).e^{-k_{max2}.t})$$

where *N*_0_ is the initial population (CFU/mL), *N* is the residual population at time *t* (CFU/mL), *f* is the fraction of the initial population in a major sub-population, (1 − *f*) is the fraction of the initial population in a minor sub-population (which is more resistant than the previous one), and *k*_*max*1_ and *k*_*max*2_ (h^−1^) are the specific inactivation rates of the two populations, respectively.

(2) $$\begin{array}{l} {\text{log}}_{10}(N)={\text{log}}_{10}((10^{{\text{log}}_{10}(N_{0})}-10^{{\text{log}}_{10}(N_{res})}).e^{-k_{max}.t}.\\ \;\left(\frac{e^{-k_{max}.S_{l}}}{1+(e^{-k_{max}.S_{l}}-1).e^{-k_{max}.t}}\right))+10^{{\text{log}}_{10}(N_{res})} \end{array}$$

where *N, N*_0_, and *K*_*max*_ have identical meaning as that reported in Equation 1, *N*_*res*_ is the residual population (CFU/mL), and *S*_*l*_ (h) is the parameter that represents the shoulder length.

(3) $${\text{log}}_{10}(N)=\frac{{\text{log}}_{10}(N_{0})}{\left(1+10^{\alpha }\right)}(10^{-{\left(\frac{t}{\delta_{1}}\right)}^{p+\alpha }}+10^{-{\left(\frac{t}{\delta 2}\right)}^{p}})$$

where *N, N*_0_, and *t* have identical meaning as that reported previously, *p* is an adimensional shape parameter, α is the ratio of the fraction of sub-population 1 to the fraction of the sub-population 2 at time 0, δ_1_ is the time (h) required for the first decimal reduction of sub-population 1, δ_2_ is the time (h) needed for the first decimal reduction of sub-population 2.

The detection limit (DL) was fixed to 1. In order to assess the goodness of fit of each model, the sum of square error (SSE), the root mean square error (RMSE), the adjusted coefficient of determination (adj-R^2^) were used.

### Survival and L-malic acid degradation in synthetic wine

The strains *Lb. plantarum* GT1 and LT11 were selected on the basis of their acid and ethanol stress response with the purpose to investigate the effect of acid and ethanol adaptation on the survival and consumption of L-malic acid in a model system at 14% ethanol and pH 3.5 (Synthetic Wine, SW). The synthetic wine was prepared as described by Bravo-Ferrada et al. (2013) and inoculated (about 1 × 10^8^ CFU/mL) with *Lb. plantarum* GT1 or LT11 preadapted in MRS containing 2% of ethanol or in MRS at pH 5.0 or in MRS BC. Cells were recovered in the mid-exponential phase as well as at the beginning of the stationary phase and then inoculated in SW. Microbial growth, pH and L-malic acid concentration were monitored during the incubation period (15 days at 20°C). Analogous experiments were performed using non-adapted cells as control.

## Results

### Screening assay

**Figure 1:**
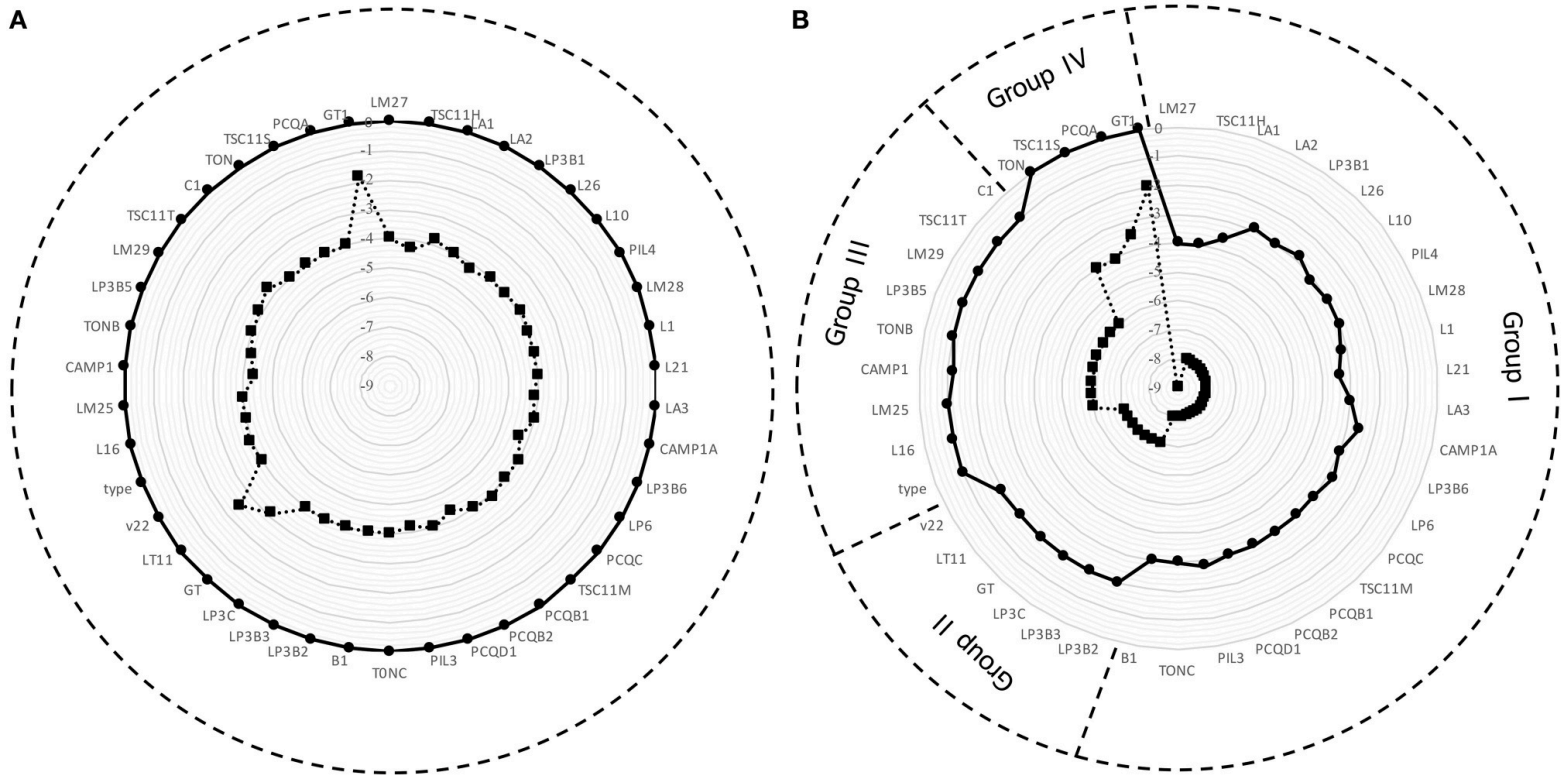
**Survival of 42 strains of *Lb. plantarum* after 2 h of exposure (A)** to 10% (•) and 14% (■) of ethanol and **(B)** to pH 3.5 (•) and pH 3.0 (■).

Figure 1 displays the survival of 42 strains of *Lactobacillus plantarum* evaluated in Ringer's solution (RS) containing 10 or 14% of ethanol (Figure 1A) and in RS acidified at pH 3.5 or 3.0 (Figure 1B). A higher variability among strains resulted from the exposure to low pH than that to ethanol. Specifically, the exposure at 10% of ethanol did not affect the survival, whereas in presence of 14% of ethanol a reduction of about 4 Log CFU/mL was observed for the assayed strains, with the exception of v22 and GT1, which were inhibited to a lower extent. A different scenario was observed when the strains were exposed to low pH (Figure 1B). In this case, *Lb. plantarum* strains were divided into 4 groups, arbitrarily individuated on the basis of the ability to survive in acid condition: group I collected 23 strains with high susceptibility to the pH; group II gathered 6 strains with a medium-high susceptibility; group III collected 9 strains with a moderate sensitivity; group IV convened 4 strains with the lowest susceptibility. On the basis of their different response to ethanol and acid stress, 10 strains (LM27, TSC11H, LM28, LP6, LT11, v22, LM25, LM29, PCQA, and GT1), randomly chosen from each group, were selected and used in the following experiments.

### Effect of ethanol and low pH on the growth of *Lb. plantarum*

**Figure 2:**
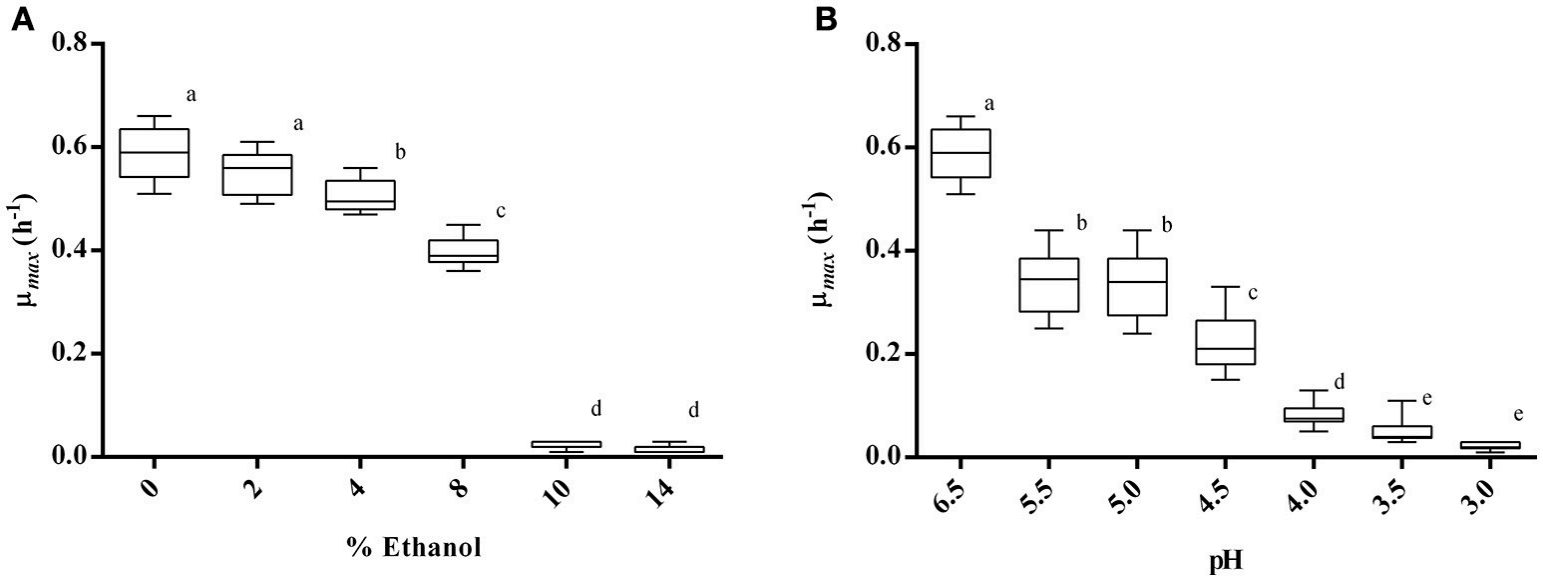
**Maximum specific growth rate (μ_*max*_) of 10 *Lb. plantarum* strains cultivated in MRS broth containing ethanol at different concentrations (A)** and in MRS broth at different pH values **(B)**. Groups with different letters are significantly different (*p* < 0.05).

The effect of different concentrations of ethanol on the growth of 10 strains of *Lb. plantarum* was tested. Results (Figure 2A) showed that no strain was able to grow in MRS containing 10 or 14% of ethanol, whereas all strains were able to grow up to 8%. However, the maximum specific growth rate (μ_*max*_) resulted significantly reduced (*p* < 0.05) when strains were cultivated in MRS containing 8% of ethanol, and a significant reduction was also appreciated in presence of 4%. Contrarily, the growth was little affected by the presence of 2% of ethanol, as evidenced by the μ_*max*_ (0.55 ± 0.04) which did not vary significantly (*p* > 0.05) if compared with the control.

The effect of low pH on the growth of the selected *Lb*. *plantarum* strains is reported in Figure 2B. No strain was able to grow at pH 3.0, and a slight growth was appreciated at pH 3.5 and 4.0. Starting from pH 4.5, most of the strains showed the ability to grow, even if the corresponding μ_*max*_ (0.22) was significantly lower than the control. The kinetic of growth at pH 5.5 or 5.0 showed in both cases a reduction of the μ_*max*_ of about 2-fold in comparison with the control (pH 6.5), but between the two conditions (pH 5.5 and pH 5.0) no significant difference was recognized (*p* > 0.05).

### Effect of long-term adaptation to ethanol and low pH on the growth of *Lb. plantarum*

The 10 selected strains of *Lb. plantarum*, pre-cultivated in MRS broth containing 2 or 8% of ethanol until the stationary phase (long-term adaptation), were subsequently cultivated in the same medium containing 8, 10, or 14% of ethanol to investigate if a non-lethal alcohol concentration was able to improve the cellular growth in presence of a high ethanol concentration. Results showed that the growth in MRS containing 10 or 14% of ethanol was inhibited independently by the preadaptation to 2 or 8% (data non shown). Strains preadapted to 8% of ethanol and then transferred in the same medium with the same alcohol concentration showed identical kinetics of growth with the controls (data not shown). Instead, a higher growth than that obtained in control conditions was observed when the strains were cultivated with 8% of ethanol after a preadaptation to 2%. Results are reported in Figure S1A, where a significant (*p* < 0.05) higher μ_*max*_ of preadapted strains was detected compared with their controls. This datum was particularly evident for the preadapted strains GT1 and LT11, having μ_*max*_ values 1.4- and 1.3-fold higher than those of their controls, respectively.

The impact of the long-term adaptation to low pH (5.0 and 4.0) on the growth at pH 3.5 or 3.0 of the selected *Lb. plantarum* strains was also evaluated. Results showed that the growth in MRS at pH 3.0 was inhibited independently by the preadaptation to pH 4.0 or 5.0 (data non shown). Moreover, strains preadapted to pH 4.0 (data non shown) or 5.0 (Figure S1B) and then transferred in the same medium at pH 3.5 showed similar kinetics of growth then their controls. A sensitive increment of the μ_*max*_ was detected only for the strain GT1 preadapted to pH 5.0 and subsequently cultivated in MRS at pH 3.5.

### Effect of sub-optimal pH and ethanol concentration on the growth of *Lb. plantarum* GT1 and LT11

On the basis of previous results, sub-optimal conditions (pH 5.0 or ethanol 2%) and their combination (BC) were chosen to assess the effect on the growth of selected strains GT1 and LT11. Results (Figure S2) highlighted that the two strains had similar behaviors in the assayed growth conditions. Conversely, significant differences in the maximum specific growth rate (μ_*max*_) were detected depending on the different growth conditions. The presence of 2% of ethanol did not affect the growth of both strains (Figures S2A,B), as showed by μ_*max*_ values substantially similar to those detected in the respective controls. The growth was instead significantly affected when GT1 and LT11 were cultivated at pH 5.0 or in BC. In fact, both situations caused a significant μ_*max*_ decrease (*p* < 0.05) which assumed values of 1.6- and 2.5-fold lower then the controls, respectively.

### Effect of long-term adaptation to ethanol and low pH on the survival of *Lb. plantarum* GT1 and LT11

**Figure 3:**
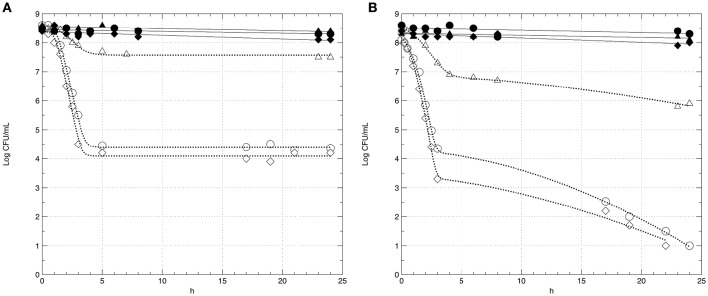
**Survival curves of *Lb. plantarum* GT1 (A)** and LT11 **(B)** exposed to ethanol 14% into RS. Symbols represent the mean of experimental data obtained by three independent experiments and lines represent the models carried out with the add-in GInaFiT. Full symbols and continuous lines refer to cell recovered in the stationary phase; empty symbols and dotted lines indicate cells withdrawn in the exponential growth phase; circles (•, ◦) indicate cells non-long-term preadapted to ethanol (0%) prior to exposure; triangles (▴, Δ) refer to cells submitted to a long-term adaptation to ethanol 2%; diamonds (♦, ♢) indicate cells preadapted to BC (ethanol 2% and pH 5.0).

**Figure 4:**
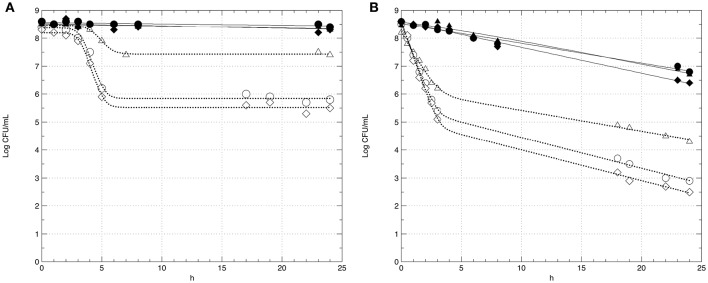
**Survival curves of *Lb. plantarum* strains GT1 (A)** and LT11 **(B)** exposed to pH 3.5 into RS. Symbols represent the mean of experimental data obtained by three independent experiments and lines represent the models carried out with the add-in GInaFiT. Full symbols and continuous lines refer to cell recovered in the stationary phase; empty symbols and dotted lines indicate cells withdrawn in the exponential growth phase; circles (•, ◦) indicate cells non-long-term preadapted to acid (pH 6.5) prior to exposure; triangles (▴, Δ) refer to cells submitted to a long-term adaptation at pH 5.0; diamonds (♦, ♢) indicate cells preadapted to BC (ethanol 2% and pH 5.0).

In this step, the survival of strains GT1 and LT11 in Ringer's solution (RS) containing 14% of ethanol after a long-term adaptation to ethanol 2% or to BC (pH 5.0 and ethanol 2%) was evaluated. The survival of preadapted cells was compared with the survival of non-preadapted cells. Moreover, the impact of the physiologic state (exponential phase *vs* stationary phase) of cells on the survival in ethanol 14% was evaluated (Figure 3). Analogously, the survival of GT1 and LT11 in RS at pH 3.5 after a long-term adaptation to pH 5.0 or to BC was compared with the survival of non-preadapted cells used in both exponential and stationary phase (Figure 4).

In all the analyzed conditions, the physiological state had a strong influence on the survival of the two strains. In fact, preadapted cells showed the same behavior of non-preadapted cells only when the cultures were recovered at the beginning of the stationary phase, and this fact concerned both ethanol and pH tests (Figures 3, 4). In detail, regardless to the ethanol sensitivity, the microbial load of the strains GT1 and LT11 withdrawn in the stationary phase did not decrease after 24 h of incubation in RS containing ethanol 14% (Figure 3). On the other hand, when strains collected in the stationary phase were tested at pH 3.5 (Figure 4), our results showed a decrease of about 1 Log CFU/mL only for LT11 (Figure 4B).

Conversely, cells recovered in the exponential phase showed a survival decay, which was more marked in the case of non-preadapted cells or cells preadapted to BC. Additionally, a different trend in the survival curves was appreciated between the two strains preadapted in different conditions. In fact, the exposure to 14% of ethanol or to pH 3.5 caused the lowest cell decrease (about 1 Log CFU/mL in both conditions) for GT1 preadapted in ethanol 2% or at pH 5, respectively, whilst the corresponding non-preadapted strain and that preadapted to BC showed a marked reduction in 14% ethanol (about 4 Log CFU/mL) and a minor decrease at pH 3.5 (about 2.5 Log CFU/mL) (Figures 3A, 4A).

The strain LT11 withdrawn in the exponential phase and exposed to 14% of ethanol or to pH 3.5 showed a different behavior compared to GT1. In detail, cells of LT11 had an overall microbial reduction of about 2.5 Log CFU/mL when preadapted in ethanol 2% and exposed to 14% of alcohol, and the highest drop was appreciated in the first 3 h of incubation (Figure 3B). LT11 preadapted to pH 5 and exposed to pH 3.5 had an overall microbial drop of about 4 Log CFU/mL (Figure 4B).

LT11 non-preadapted or preadapted to BC was strongly injured by the presence of high alcohol concentration or by the low pH when collected in the exponential phase, as highlighted by a decrease of about 7.5 or 5.5 Log CFU/mL registered at the end of the incubation with 14% of ethanol or at pH 3.5, respectively (Figures 3B, 4B).

The survival data were modeled using GInaFiT tool (Tables 1, 2). Out of 9 inactivation models fitted with the GInaFiT tool, 3 showed the finest statistical parameters (SSE, RMSE, and adj-R^2^). Specifically, considering the survival in the medium containing 14% of ethanol, the Log-linear model with shoulder and tail and the double Weibull model were the two mathematical functions that better described the survival of strains GT1 (Figure 3A) and LT11 (Figure 3B), respectively. Regarding to the survival in the medium at pH 3.5, the Log-linear model with shoulder and tail and the biphasic linear model were individuated as the best functions to describe the behavior of GT1 (Figure 4A) and LT11 (Figure 4B), respectively.

**Table 1:** **Survival kinetic parameters of strains GT1 and LT11 withdrawn in the exponential phase and evaluated in RS containing ethanol 14% after non-preadaptation (Non-preadapt), preadaptation ethanol 2% (Preadapt_Et2) or preadaptation to BC (ethanol 2% and pH 5.0) (Preadapt_2/5)**.

**Strain**	**Preadapt/Non-preadapt**	**Model**	**Log(*N_0_*)**	**Log(*N_res*)**	***S_*l*_***	***K_*max*_***	**α**	***δ_1_***	***p***	***δ_2_***	**4D**	**SSE**	**RMSE**	**Adj-R^2^**
GT1	Non-preadapt	Log_lin+S+T	8.6	4.4	1.18	4.15					±3.8	0.039	0.075	0.998
		SE	0.0	0.0	0.07	0.26								
	Preadapt_Et2	Log_lin+S+T	8.5	7.5	1.86	1.74					>24	0.040	0.067	0.974
		SE	0.0	0.0	0.23	0.43								
	Preadapt_2/5	Log_lin+S+T	8.4	4.1	1.11	4.65					±3.4	0.224	0.158	0.993
		SE	0.1	0.1	0.12	0.37								
LT11	Non-preadapt	Double_Weibull	8.1				3.78	1.3	1.8	12.4	±5.5	0.072	0.095	0.999
		SE	0.1				0.13	0.1	0.1	0.6				
	Preadapt_Et2	Double_Weibull	8.5				1.60	2.5	1.7	23.3	>24	0.029	0.064	0.996
		SE	0.0				0.06	0.1	0.2	1.1				
	Preadapt_2/5	Double_Weibull	8.1				4.74	1.1	1.6	13.5	±2.6	0.174	0.158	0.997
		SE	0.1				0.27	0.1	0.2	1.3				

*Log_lin+S+T, Log linear model with shoulder and tail; N_0_, initial population (Log CFU/mL); N_res, residual population (Log CFU/mL); S_l_, shoulder length (h); K_max_, specific inactivation rate; α is the ratio of the fraction of sub-population 1 to the fraction of the sub-population 2 at time 0, p, dimensionless shape parameter; δ_1_, time (h) required for the first decimal reduction of subpopulation 1; δ_2_, time (h) required for the first decimal reduction of subpopulation 2; 4D, time (h) required for the population reduction of 4 Log CFU/mL; SSE, sum of square error; RMSE, root mean square error; Adj-R^2^, adjusted coefficient of determination*.

**Table 2:** **Survival kinetic parameters of strains GT1 and LT11 withdrawn in the exponential phase and evaluated in RS at pH 3.5 after non-preadaptation (Non-preadapt), preadaptation to pH 5.0 (Preadapt_5) or preadaptation to BC (ethanol 2% and pH 5.0) (Preadapt_2/5)**.

**Strain**	**Preadapt/Non-preadapt**	**Model**	**Log(*N_0_*)**	**Log(*N_res*)**	***S_*l*_***	***K_*max*_/K*_*max*1_**	***K*_*max*2_**	***f***	**4D**	**SSE**	**RMSE**	**Adj-R^2^**
GT1	Non-preadapt	Log_lin+S+T	8.4	5.8	3.21	2.90			>24	0.138	0.152	0.984
		SE	0.1	0.1	0.25	0.53						
	Preadapt_5	Log_lin+S+T	8.5	7.4	4.31	2.03			>24	0.017	0.056	0.987
		SE	0.0	0.0	0.18	0.49						
	Preadapt_2/5	Log_lin+S+T	8.2	5.5	3.12	2.99			>24	0.116	0.139	0.988
		SE	0.1	0.1	0.22	0.46						
LT11	Non-preadapt	Biphasic	8.5			2.63	0.25	0.9990	±9.4	0.054	0.088	0.998
		SE	0.1			0.13	0.02	0.0005				
	Preadapt_5	Biphasic	8.2			1.72	0.17	0.9913	>24	0.047	0.082	0.997
		SE	0.1			0.14	0.03	0.0047				
	Preadapt_2/5	Biphasic	8.6			2.84	0.25	0.9997	±4.8	0.095	0.116	0.997
		SE	0.1			0.15	0.04	0.0002				

*Log_lin+S+T, Log linear model with shoulder and tail; N_0_, initial population (Log CFU/mL); N_res, residual population (Log CFU/mL); S_l_, shoulder length (h); K_max_, specific inactivation rate (h^−1^); K_max1_ and K_max2_, specific inactivation rates (h^−1^) of the subpopulation 1 and 2 respectively; f, fraction of the initial population in major subpopulation; 4D, time (h) required for the population reduction of 4 Log CFU/mL; SSE, sum of square error; RMSE, root mean square error; Adj-R^2^, adjusted coefficient of determination*.

The kinetic parameters obtained by the models highlighted different responses of the two strains to 14% of ethanol (Table 1). In detail, during the first hours of exposure to ethanol, a population fraction of non-preadapted cells of LT11 was reduced of 4 Log units (4D) in 5.5 h, about 1.5-fold more than GT1 (3.8 h). A further reduction of LT11 was observable when the exposure was prolonged. In fact, after 24 h of exposure to 14% of ethanol, the strain LT11 was no more detectable, whereas the microbial load of GT1 was about 4 Log CFU/mL.

The datum regarding the preadaptation effect in 2% of ethanol on the survival of LT11 and GT1 was particularly interesting. The results (Table 1) showed that when the strain LT11 was preadapted to ethanol, the 4D value was > 24 h, and the kinetic parameters δ_1_ and δ_2_ were 2-fold higher than those of non-preadapted cells. These parameters represent the time to obtain a decimal reduction of the first (δ_1_) and second (δ_2_) fraction of the microbial population. The most important improvement of the kinetic parameters was also observed in the model representing the survival of GT1 after ethanol-adaptation. Among all, the Log(*N_res*) parameter, that represents the “tail” of model, resulted about 2-fold higher than that of non-preadapted cells. Moreover, the microbial load corresponding to the tail was reduced of 1 Log unit compared with the microbial load at the beginning of the exposure (Log_*N*_0_).

The long-term adaptation to BC did not positively affect the kinetic parameters. In fact, the 4D value and the Log(*N_res*) parameters for both strains were similar or even lower than those observed for non-preadapted cells.

Considering the survival at low pH, the results regarding the kinetic parameters (Table 2) highlighted that non-preadapted LT11 had a decay of the survival with a *k*_*max*1_ of 2.63 and a *k*_*max*2_ of 0.25. These parameters were substantially reduced (−1.5-fold) for cells preadapted to pH 5.0. A similar behavior was observed for the strain GT1. In detail, when the cells adapted to pH 5.0 where exposed to pH 3.5, the kinetic parameter representing the shoulder (*S*_*l*_) of the curve increased of 1.3 units compared to that recorded for non-preadapted cells. Moreover, the *k*_*max*_ value was reduced of about 1.5 units for the preadapted cells compared to non-preadapted ones. The kinetic parameter regarding the residual population (Log_*Nres*) after 24 h of exposure to pH 3.5 was also noticeable. In this case, the microbial load was higher than 7 Log CFU/mL for sub-optimal pH preadapted cells and lower than 6 Log CFU/mL for non-preadapted cells. Finally, no improvement was observed when the strains were preadatpted to BC.

### Survival of *Lb. plantarum* GT1 and LT11 and L-malic acid degradation in synthetic wine

**Figure 5:**
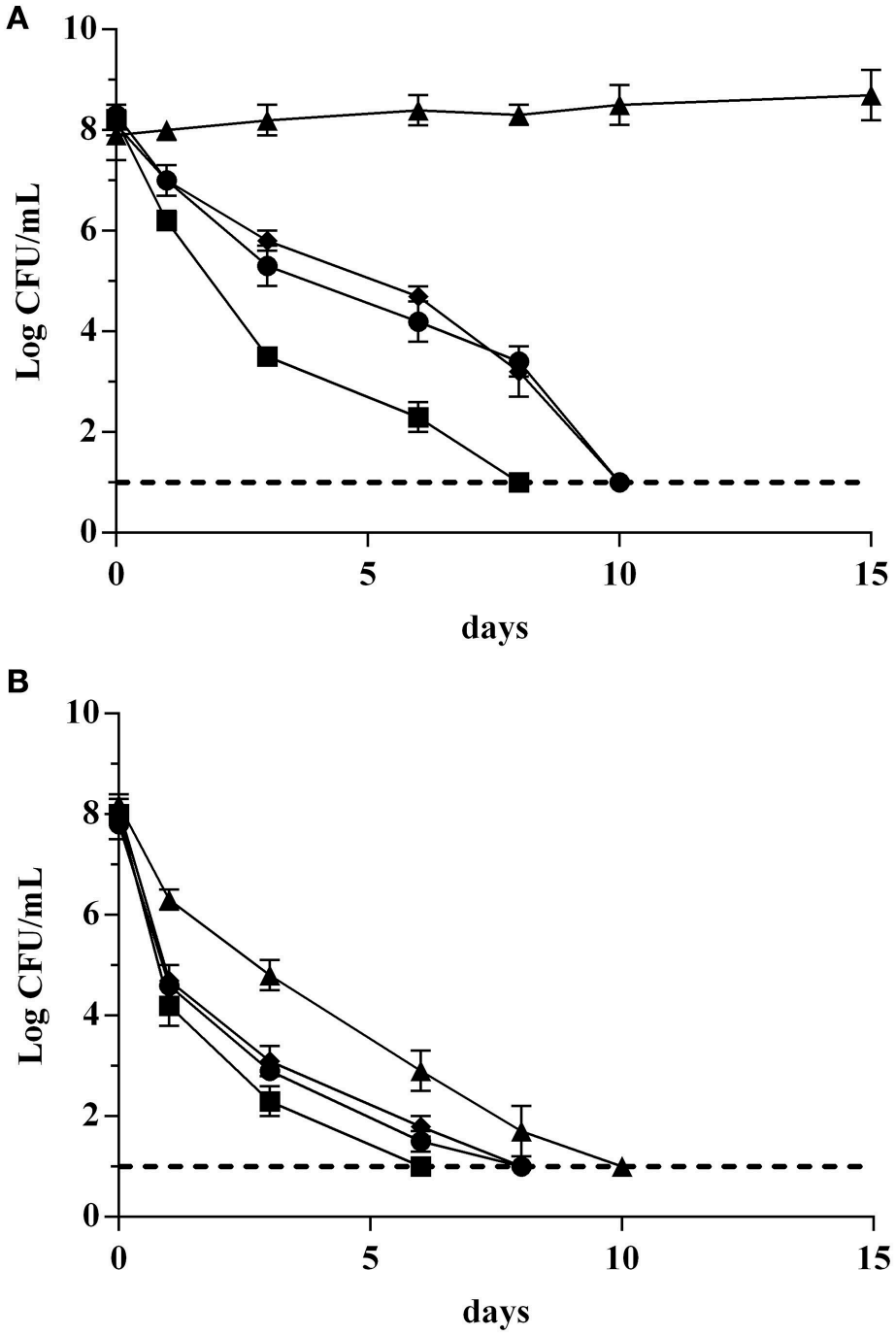
**Survival curves of *Lb. plantarum* GT1 (A)** and *Lb. plantarum* LT11 **(B)** in synthetic wine (SW) after recovering in the stationary phase. Different symbols indicate cells non-preadapted (•) or long-term preadapted to ethanol 2% (■), pH 5.0 (▴), or to BC (ethanol 2% and pH 5.0) (♦).

In this final step, a synthetic wine (SW) at 14% ethanol and pH 3.5 was used to assess the behavior of the strains GT1 and LT11 preadapted in MRS containing ethanol 2%, or acidified at pH 5.0, or BC. Cells were collected in the exponential phase or at the beginning of the stationary phase and incubated in SW for 15 days at 20°C. As expected, both strains recovered in the exponential phase showed a very high survival decay in SW, regardless of the preadaptation conditions (data not shown). Better performances were appreciated when the cells were collected at the beginning of the stationary phase (Figure 5). In detail, considering the strain GT1 collected at the beginning of the stationary phase, the preadaptation to pH 5 significantly improved the survival in SW (Figure 5A). In fact, after 15 days of incubation, the microbial load was substantially unaffected (about 10^8^ Log CFU/mL). However, an improvement of the survival in comparison with non-preadapted cells was also observed for cells preadapted to BC, whereas cells preadapted to ethanol 2% resulted more sensitive than the control. Considering the tests carried out on LT11, the strain highlighted a complete decay in spite of preadapation conditions (Figure 5B).

**Figure 6:**
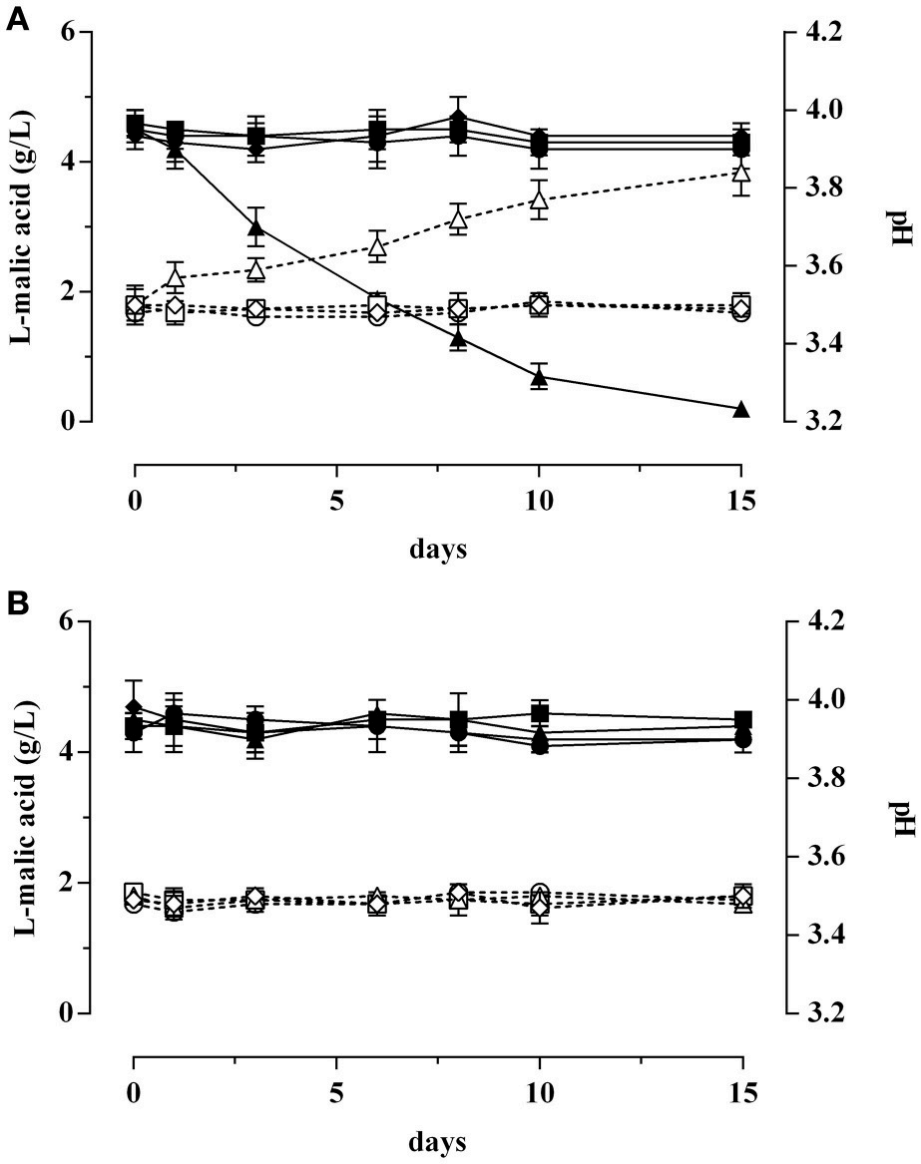
**L-malic acid degradation (full symbols) and pH evolution (empty symbols) in synthetic wine (SW) inoculated with *Lb. plantarum* GT1 (A)** or with *Lb. plantarum* LT11 **(B)**. Cells were collected in the stationary phase after non-long-term preadaptation (•, ◦), or after a long-term preadaptation to ethanol 2% (■, □), to acid (pH 5.0) (▴, Δ), or to BC (ethanol 2% and pH 5.0) (♦, ♢).

The trend of L-malic acid concentration and pH of the SW inoculated with GT1 recovered at the beginning of the stationary phase and preadapted to ethanol 2%, or to pH 5.0, or to BC is displayed in Figure 6A. The results revealed that the L-malic acid concentration and the pH values remained unaffected when the SW was inoculated with non-preadapted GT1, with GT1 cells preadapted to ethanol 2% or with cells preadapted to BC. On the contrary, the L-malic acid resulted completely degraded after 15 days of incubation with acid-preadapted GT1. Moreover, the pH value increased of about 0.3 units only when the SW was inoculated with acid-preadapted cells.

Instead, no changes were observed in L-malic acid concentration and in pH values when preadapted or non-preadapted LT11 cells were inoculated in SW (Figure 6B).

## Discussion

The success of *Lb. plantarum* as starter in the MLF depends mainly on the strain used, whose metabolic activities are strongly linked to the ability to adapt to the main hurdles of wine (mainly high content of ethanol and low pH). As reported by several Authors (duToit et al., 2011; Tremonte et al., 2017) both ethanol and pH tolerance are strain-dependent features. So, in our study 42 *Lb. plantarum* strains were preliminary screened for their ability to survive at different ethanol and pH values. In detail, conditions characterizing wines from cool (10% of ethanol and pH 3.0) and warm (14% of ethanol and pH 3.5) climates (Henick-Kling, 1993; Liu, 2002) were chosen for the preliminary screening, which highlighted that the cell survival is strongly influenced by the highest ethanol level (14%) and, more markedly, by the low pH (both pH 3.0 and 3.5). Our study also allowed to individuate limit growth values for 10 selected strains (pH 3.5 and ethanol 8%). The inability to grow in the presence of more than 8% of ethanol was in agreement with several Authors (Guerzoni et al., 1995; Berbegal et al., 2016), even if other Authors (G-Alegría et al., 2004; Iorizzo et al., 2016) reported that some strains of *Lb. plantarum* were able to grow not only in the presence of 13% ethanol, but also at pH values ranging from 3.2 to 3.5.

In our opinion, the most important result was obtained from the data related to the long-term adaptation to sub-optimal pH and ethanol content. In fact, specific strains showed an improvement in the μ_*max*_ when exposed to acid (pH 3.5) or ethanol (8%) stress conditions after a long-term adaptation to pH 5.0 or in ethanol 2%, respectively. Similar results were already reported for *Oenococcus oeni* by G-Alegría et al. (2004), who evidenced the positive effect of low ethanol content (3–4%) on the growth of strains belonging to this species. However, the favorable impact of the log-term adaptation to ethanol 2% was not observed when the strains were cultivated at higher ethanol content (10 or 14%).

On the contrary, the survival ability of specific *Lb. plantarum* strains (GT1 and LT11) in media with ethanol 14% or acidified at pH 3.5 was positively affected by the long-term adaptation to ethanol 2% and at pH 5.0, respectively. So, as also highlighted by several Authors (Broadbent et al., 2010; Bravo-Ferrada et al., 2013; Tremonte et al., 2016b) we found that culture adaptation to sub-optimal stress conditions led to an improvement of tolerance to the same stress but in more extreme conditions.

Interestingly, van de Guchte et al. (2002) stated that cells exposed to a sub-lethal stress could improve the resistance against other type of stress (cross-protection). Moreover, in a recent study Huang et al. (2015) reported that the exposure of *Lb. plantarum* ZDY2013 to acid stress induced cross-protection against oxidative stress. Recently, van Bokhorst-van de Veen et al. (2011) showed that the long-term adapted cells of *Lb. plantarum* to ethanol 8%, lead a cross-protective effect versus high temperatures but not versus other stress conditions including acid. Similarly, we found that the sole ethanol adaptation or the concurrent adaptation to 2% of ethanol and pH 5.0 did not improve the survival of the assayed strain in synthetic wine. These results evidence that the adaptation to low levels of ethanol does not induce a cross protective effect against low pH. On the contrary, the preadaptation to pH 5 strongly improved the survival of GT1 in synthetic wine, that is, a cross protective effect against high ethanol content.

Different molecular mechanisms were suggested for the response of *Lb. plantarum* to long-term exposure to sub-optimal pH or to ethanol. With reference to the acid stress response, a large spectrum of different cellular functions was proposed in the maintenance of the intracellular pH homeostasis (pH_i_) and of the proton-translocation. They include the F_1_F_0_-ATPase complex, the arginine deaminase (ADI), the glutamate decarboxylase (GAD) pathways and the expression of general stress proteins (GSPs) and molecular chaperones that repair (e.g., dnaK, groES, groEL) or degrade (e.g., ClpL, ClpC, ClpP) damaged DNA and proteins (van de Guchte et al., 2002; De Angelis and Gobbetti, 2011). Regarding the ethanol response, several Authors reported that lactic acid bacteria are able to rearrange the membrane lipid composition (Bravo-Ferrada et al., 2015) or the citoplasmatic and membrane protein pattern which can also involve the expression of small heat shock proteins (Silveira et al., 2004; Spano et al., 2004; Fiocco et al., 2007).

Previous data should be taken into account during the selection or the use of MLF starter. In fact, several Authors suggest to add MLF starter at the beginning of alcoholic fermentation with the aim to allow a gradual adaptation of the starter to the increasing alcohol concentration in the wine (Jussier et al., 2006; Zapparoli et al., 2009; Bartowsky et al., 2015; Tristezza et al., 2016). Instead, our data suggest that a preadaptation to a sub-optimal pH value is a valuable tool to improve the survival of oenological *Lb. plantarum* strains.

Moreover, the survival kinetic parameters resulted highly affected by the physiological state of cells. In fact, the cells recovered in the stationary phase showed a higher tolerance to stressors (ethanol 14% and pH 3.5) than that exhibited by cells collected in the exponential phase.

In this context, it is known that LAB generally display an increase in stress resistance during the stationary phase (van de Guchte et al., 2002; Zotta et al., 2008). This behavior could be attributed to a complex stress response mechanism that involves the synthesis of several general stress proteins to cope the starvation stress (van de Guchte et al., 2002).

On the other hand, cells recovered in the exponential phase showed a decay of survival with a non-linear kinetic mainly strain-dependent. In fact, the strain GT1 displayed survival curves compatible with the log-linear model with shoulder and tail (Geeraerd et al., 2000) when exposed to ethanol 14% or at pH 3.5. Instead, the strain LT11 produced curves with a shape compatible with the biphasic model (Cerf, 1977) in the presence of acid stress conditions (pH 3.5) and curves that show a trend matching the Double Weibull model (Coroller et al., 2006) in presence of ethanol stress conditions (14%). These results are in agreement with other studies highlighting that bacterial strains display different non-thermal inactivation curves depending on several factors including the physiological state (exponential or stationary phase) and the type of stress (Phan-Thanh et al., 2000; Greenacre et al., 2003; Coroller et al., 2006; Hajmeer et al., 2006; Pragalaki et al., 2013). These Authors evidenced that the shape of curves could change according to the intensity of the stress and to the adaptation conditions before the stress exposition. Instead, in our study we did not observe differences between the shape of the survival curves related to long-term adapted and non-adapted strains.

Furthermore, a high efficiency, in terms of acid malic consumption and pH increase was observed in acid-adapted cells recovered in the stationary phase. This last observation is in agreement with Miller et al. (2011) who described a relation between the low pH and the expression of the malolactic enzyme gene (*mle*) in *Lb. plantarum*.

Finally, it is possible to state that the use of *Lb. plantarum* strains long-term adapted to sub-optimal pH values and collected in the stationary phase could represent a valid technological strategy to optimize the course of the malolactic fermentation.

## Author contributions

MS: Experimental designing, drafting the work and revising it critically, agreement to be accountable for all aspects of the work in ensuring that questions related to the accuracy or integrity of any part of the work are appropriately investigated and resolved. GP: Design of the work, analysis and interpretation of the microbial data, and drafting the work. PT: Experimental designing, analysis and interpretation of data. LT: Analysis and interpretation of the microbial data. RC: Conception of the work, drafting the work and revising it critically. MI: Involved in experimental designing. SL: Evaluation of L-malic acid degradation. ES: Drafting the work and revising it critically.

### Conflict of interest statement

The authors declare that the research was conducted in the absence of any commercial or financial relationships that could be construed as a potential conflict of interest.
