# Supplementary material for: Sub-optimal pH Preadaptation Improves the Survival of Lactobacillus plantarum Strains and the Malic Acid Consumption in Wine-Like Medium
Source: Front Microbiol. 2017 Mar 22;8:470. doi: 10.3389/fmicb.2017.00470 (PMC5360758; doi:10.3389/fmicb.2017.00470)
Supplement: Supplementary file 1 [file Image1.PDF]

Supplementary Material

Sub-optimal pH preadaptation improves the survival of *Lactobacillus plantarum* strains and the malic acid consumption in wine-like medium

Mariantonietta Succi*, Gianfranco Pannella, Patrizio Tremonte, Luca Tipaldi, Raffaele Coppola, Massimo Iorizzo, Silvia Jane Lombardi, Elena Sorrentino

*** Correspondence:** Mariantonietta Succi: succi@unimol.it

Supplementary Figure 1

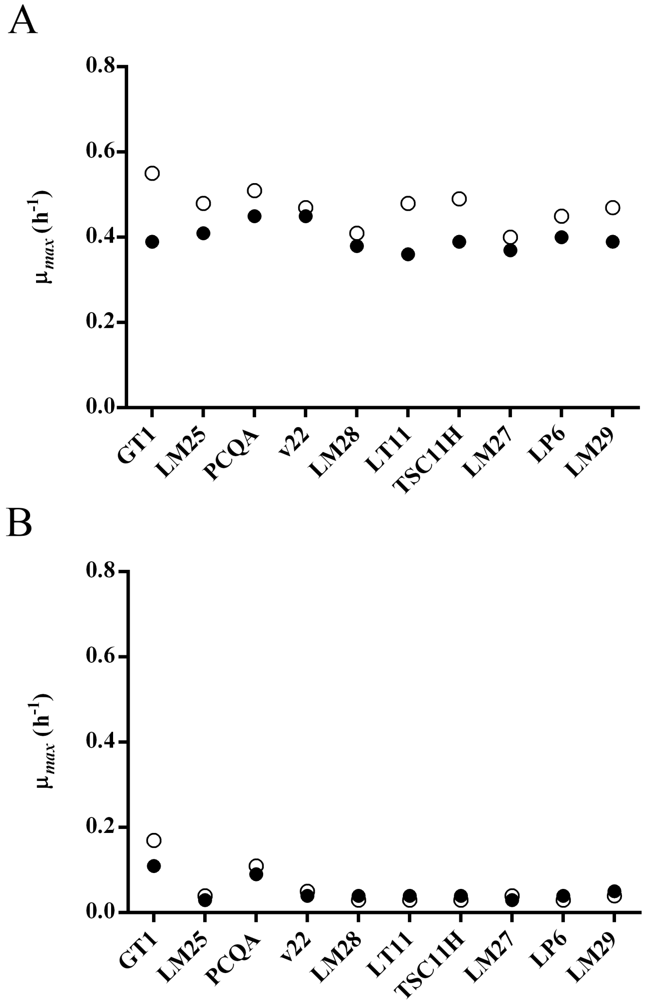

Figure S1. Maximum specific growth rate (μ_{max}) of 10 *Lb. plantarum* strains cultivated in MRS broth containing ethanol 8% after a long-term adaptation to ethanol 2% (○) and no preadaptation (●) **(A)**, and in MRS broth at pH 3.5 after a long-term adaptation at pH 5.0 (○) and no preadaptation (●) **(B)**.
