# Supplementary material for: Sub-optimal pH Preadaptation Improves the Survival of Lactobacillus plantarum Strains and the Malic Acid Consumption in Wine-Like Medium
Source: Front Microbiol. 2017 Mar 22;8:470. doi: 10.3389/fmicb.2017.00470 (PMC5360758; doi:10.3389/fmicb.2017.00470)
Supplement: Supplementary file 2 [file Image2.PDF]

Supplementary Material

Sub-optimal pH preadaptation improves the survival of *Lactobacillus plantarum* strains and the malic acid consumption in wine-like medium

Mariantonietta Succi*, Gianfranco Pannella, Patrizio Tremonte, Luca Tipaldi, Raffaele Coppola, Massimo Iorizzo, Silvia Jane Lombardi, Elena Sorrentino

*** Correspondence:** Mariantonietta Succi: succi@unimol.it

Supplementary Figure 2

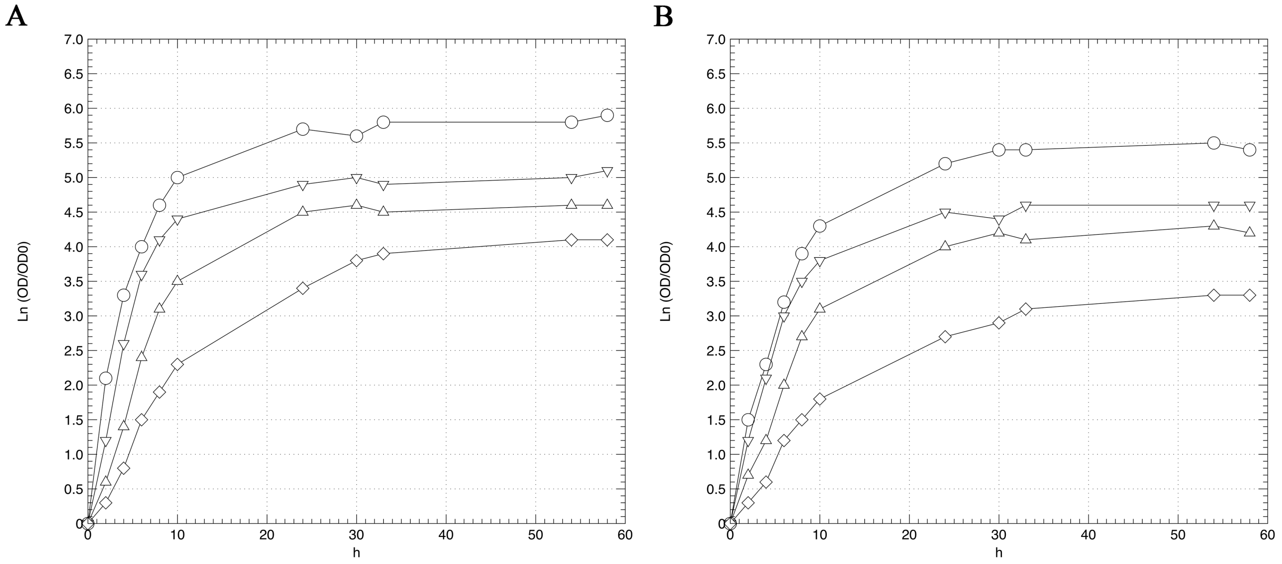

Figure S2. Growth curves of *Lb. plantarum* GT1 (**A**) and *Lb. plantarum* LT11 (**B**) cultivated in MRS broth at pH 6.5 (○), pH 5.0 (△), with ethanol 2% (▽), or with BC (ethanol 2%, pH 5.0) (◇).
